# Dietary and agricultural adaptations to drought among smallholder farmers in South Africa: A qualitative study

**DOI:** 10.1016/j.wace.2022.100413

**Published:** 2022-03

**Authors:** Poppy Hawkins, Wendy Geza, Tafadzwanashe Mabhaudhi, Catherine Sutherland, Kevin Queenan, Alan Dangour, Pauline Scheelbeek

**Affiliations:** a Centre for Agriculture, Food and Environmental Management (CAFEM), University of Hertfordshire, United Kingdom; b School of Agricultural, Earth and Environmental Sciences, University of KwaZulu-Natal, South Africa; c School of Built Environment & Development Studies, University of KwaZulu-Natal, South Africa; d Department of Pathobiology and Population Sciences, Royal Veterinary College, United Kingdom; e Centre on Climate Change & Planetary Health, London School of Hygiene & Tropical Medicine, United Kingdom

**Keywords:** South Africa, Climate change, Climate extremes, Food consumption, Food insecurity, Community resilience, Vulnerability

## Abstract

Building resilience to environmental change is an integral part of long-term climate adaptation planning and local policy. There is an increased understanding of the impact of climate change on global crop production however, little focus has been given to local adaptation pathways and rural smallholder community responses, especially regarding food security. It is becoming increasingly evident that local level decision-making plays a vital role in reducing vulnerability to environmental change. This research aimed to qualitatively investigate coping and adaptive strategies adopted by smallholder farming households to respond to the impacts of drought in rural KwaZulu-Natal, South Africa. Focus group discussions (n = 7) consisting of 5–9 participants and individual interviews (n = 9) using pre-tested topic guides, involving a total of 57 adults were conducted in rural areas of drought-affected districts: Msinga, Richmond and Umbumbulu of KwaZulu-Natal, in July 2018. The data were analysed using thematic analysis in NVivo 12. Thematic analysis identified three principal themes: 1. Perceived effects of droughts on the local food system and diets; 2. Current coping strategies; and 3. Enablers for successful adaptation. All sites reported a change in food consumption habits, with the majority perceiving drought to be the main driver behind a shift from vegetable-based to starch-based diets and decreased animal source food consumption. Only short-term coping strategies were implemented across the study sites. However, knowledge of long-term adaptation strategies existed but was unattainable to most respondents. Recommendations of perceived context-specific long-term adaptation strategies that could be used at a local scale were communicated by the respondents. However, they would need external help to actualize them. A need exists to support smallholder communities’ short-term response methods to drought to achieve more holistic resilience and successful adaptation. Short-term adaptation strategies, if implemented alone, often have significant trade-offs with longer-term adaptation and building resilience. This study highlights the need for targeted, contextualised policy solutions to improve smallholder productivity during drought through a strategic combination of both short- and longer-term adaptation measures, i.e. short-term adaptation should be guided by a long-term adaptation strategy. Proper planning, including the use of climate scenarios combined with information on nutritional status, is needed to develop context-specific and transformative adaptation strategies. These strategies should aim to strengthen resilience at a local level and should be included as policy recommendations.

## Highlights

• Smallholders perceived drought as major driver of change in consumption patterns and feared adverse health impacts in the long-term.
• Evidence on nutritional status as result of drought-induced changes in crop production and consumption remains scarce.
• Short-term coping strategies were more prevalent than longer term sustainable adaptation strategies in response to drought.
• Awareness of adaptive measures is evident but communities often lack implementation capacity, leading to maladaptation.
• Community engagement and use of indigenous knowledge in co-creation of adaptation strategies is vital for improvement of stakeholder resilience.

## 1 Introduction

Climate variability and change pose major threats to global food security at the local, national and global level (Tilman and Clark, 2014; Food and Agriculture Organization of the United Nations FAO, 2016a, Food and Agriculture Organization of the United Nations FAO, 2016b; Springmann et al., 2016; Myers et al., 2017; Scheelbeek et al., 2018; Kom et al., 2020). The Intergovernmental Panel on Climate Change (IPCC) predicts, with high confidence, an increase in climatic variability, as well as an increase in frequency and severity of extreme weather and, as a result, environmental variability and change in the near future, at different scales (IPCC, 2018), a situation which is already a reality across many parts of the world (IPCC, 2012; Miyan, 2015; Connolly-Boutin and Smit, 2016). In the absence of adequate adaptation and mitigation strategies (i.e. modification of systems or activities, including those in agriculture, to reduce the risk of and/or vulnerability to a particular shock (UNFCCC, 2010)). Climate variability (CV) and climate change (CC) will severely alter growing conditions for global agriculture, and likely result in widespread food shortages, food insecurity and adverse health impacts, through social, environmental, and economic pathways (Adger et al., 2007; Campbell-Lendrum and Corvala, 2007).

The impacts of climate variability and change are highly inequitable. The poor, less resilient and most vulnerable populations, who contribute the least to greenhouse gas (GHG) emissions are often hardest-hit by the impacts of climate variability and change as they lack the capacity to recover from shocks (Campbell-Lendrum and Corvala, 2007; Costello et al., 2009; IPCC, 2014). Prolonged drought periods are among the most severe climate change-related consequences, and can have devastating impacts on agriculture, socio-economic activities and natural environments (FAO, 2008; Food and Agriculture Organization of the United Nations FAO, 2016a, Food and Agriculture Organization of the United Nations FAO, 2016b; Ali et al., 2017; FAO, IFAD, UNICEF, 2017; Bakhshandeh et al., 2018; Scheelbeek et al., 2018). The consequences of severe (i.e. lengthy) drought periods are often critical, leading to water shortages for multiple uses, negative impacts on crop production, animal production and water, sanitation and hygiene problems (and their associated ill health) in the population, especially in marginal settings (Orimoogunje, 2017). Drought is a slow-onset hazard and is often difficult to recognise, and in some cases may cause greater impacts than acute natural disasters (Orimoogunje, 2017).

The main effects on human health of drought are caused by food insecurity and reduced access to safe water (Ravindranath et al., 2005; Aggarwal and Singh, 2010; Stanke et al., 2013). However, the livelihood impacts of drought go beyond food insecurity: drought can cause substantial changes to the environment and environmental processes, increasing the severity of floods, land degradation, aridity and desertification (Wilhite and Glantz, 2009; National Drought Mitigation Centre, 2018). Individual households or communities may lose their land, livelihoods, shelter and water sources, further reducing their ability to cope with future environmental changes (Masih et al., 2014). This is especially the case for poor marginal populations – and in particular farming communities, (smallholder farmers) – whose livelihoods are dependent on natural resources and who typically have low adaptive capacity (Campbell-Lendrum and Corvala, 2007; Morton, 2007; Costello et al., 2009; IPCC, 2012). South Africa is a semi-arid country that is among the 30 driest countries in the world (Das, 2012; Department of Water Affairs, 2013; Mabhaudhi et al., 2019) and particularly prone to severe episodes of drought, related to the El Niño-Southern Oscillation (ENSO) phenomenon and a variable and changing climate (Ziervogel et al., 2014). Drought is the most prominent feature of South African weather (Rouault and Richard, 2003) and the most noteworthy natural disaster which negatively affects southern African economic, social and environmental activities (Buckland *et al.*, 2000). However, South Africa relies heavily on rain-fed agriculture for food production: sixty per cent of all ground and surface water withdrawn in the country is used for commercial agricultural irrigation (Food and Agriculture Organization of the United Nations FAO, 2012, Food and Agriculture Organization of the United Nations FAO, 2008). Smallholder farmers in this context, especially rural smallholder farmers, rely predominantly on rainfed and hand-irrigated agriculture and have limited access to relevant infrastructure or resources for irrigation (Kom et al., 2020). This, alongside South Africa's high rates of undernutrition, increasing population, unequal distribution of land and multifaceted poverty, further amplify vulnerability, especially of marginal farming communities, to environmental change (Campbell-Lendrum and Corvala, 2007; Costello et al., 2009; IPCC, 2012; Niang et al., 2014; Ziervogel et al., 2014; Claasen et al., 2016; Statistics South Africa, 2017b). In the absence of effective adaptation strategies, prolonged droughts increase the demand for irrigation and exacerbate the challenges faced by poor rural populations.

The agricultural sector in South Africa is dualistic (Hardy et al., 2011). It comprises a well-integrated, highly capitalised commercial sector with approximately 35,000 farmers, producing around 95% of agricultural output on 87% of total agricultural land (Aliber and Hart, 2009). In contrast, the smallholder sector consists of approximately 4 million farmers farming in the former homeland areas on 13% of the agricultural land of South Africa (Aliber and Hart, 2009). This division between the commercial, large-scale farming sector and the comparatively less productive smallholder sector has its origins in land and social policies of pre-democracy governments. Socio-economically, most smallholder farmers in South Africa are poor, less educated and reside in rural communities with less developed infrastructure (Statistics South Africa, 2011a, Statistics South Africa, 2011b, 2013; The World Bank, 2018). Many of these communities are governed by traditional authorities, nkosi and izinduna, who are predominantly male, while up to 80% of the active producers are females (Ngcoya and Kumarakulasingam, 2017). Most households in rural communities employ a mix of livelihoods strategies, including salaries and wages, social grants, income from business, pension remittances and natural resource extraction contributing to household income (Statistics South Africa, 2013, Statistics South Africa, 2017a, Statistics South Africa, 2017b). Agricultural activities continue to play an essential role in providing much-needed subsistence for rural communities, in the form of food and income.

Nationally, 78.5% of South African households who are involved in agriculture, do so in an attempt to secure additional food sources (Statistics South Africa, 2017a, Statistics South Africa, 2017b). The largest proportion of smallholder farming households in South Africa are located in the KwaZulu-Natal (KZN) province (25%), which is the second most highly populated province in South Africa (Statistics South Africa, 2013). KZN province is amongst the hardest-hit regions during drought periods (Evans, 2016). This is due to high vulnerability, both external (exposure) and internal (coping capabilities) (Drimie *et al.* 2005), adding to prevailing socio-economic issues such as poverty, unemployment and a lack of education and coping mechanisms, especially regarding natural disasters in rural areas (Mwaniki, 2006).

In 2016, the KZN Department of Cooperative Governance and Traditional Affairs (CoGTA) announced a formal declaration of disaster after a prolonged drought across the KZN province (South African Government, 2015), during one of the strongest El Niño events of the last 50 years (World Meteorological Organisation, 2016). This drought affected significantly on livelihoods, especially those of vulnerable, smallholder farming communities across the province. The drought resulted in smallholder farmers having to abandon their agricultural lands adjacent to their homesteads and opting for crop production in smaller plots of land in shared agricultural communal areas near water sources. A large number of smallholder farmers stopped farming around this time.

Poverty remains concentrated in previously disadvantaged areas such as the former rural homelands of South Africa where the majority of the population participate directly or indirectly in agricultural activities to sustain their livelihoods, and contribute towards the fight against food and nutrition insecurity at local and household levels to a different extent, depending on resource endowment (The World Bank, 2018). However, in these regions, climate variability and change hotspots have been observed (Schulze, 2011). And as a result, KwaZulu-Natal, for example, largely a former homeland, has some of the highest rates of poverty in the country of South Africa, highlighting the enduring effects of the apartheid era and vulnerability of rural peoples’ livelihoods to external shocks. Limited development and the marginalisation of rural communities allow for a prevalence of food and nutrition insecurity in these regions (Statistics South Africa, 2017a, Statistics South Africa, 2017b; The World Bank, 2018).

Given the importance of smallholder farming in South Africa, increased knowledge of local adaptation strategies will be vital to improve the country's overall resilience to anticipated climatic change (Niang et al., 2014). This paper presents qualitative research on drought adaptation strategies in three rural smallholder-farming communities in KwaZulu-Natal, South Africa. We aim to add to the evidence base around climate change adaptation in smallholder agriculture and guide the development of local and region-specific policies for adaptive capacity building that align with local norms and beliefs and improve future resilience to drought and environmental change.

## 2 Methods

### 2.1 Study sites

**Fig. 1:**
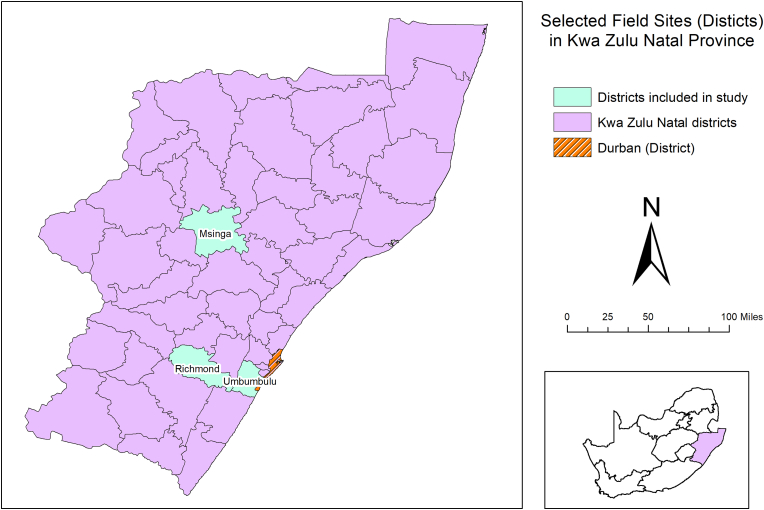
Map of KwaZulu-Natal, South Africa. Showing the locations of the districts that the study sites selected where located in.

This research focused on three sites, across KwaZulu-Natal, South Africa that are located in different climatic zones and have different agricultural and ecological zones. These sites are; Msinga (MS), Richmond (RM) and Umbumbulu (UM) (Fig. 1) and were among the most drought-affected parts of KZN during the recent drought that reached its peak during 2015/16 (World Meteorological Organisation, 2016). Msinga local municipality is located in the isolated valleys of uMzinyathi District Municipality of KwaZulu-Natal. It is one of the 20 poorest municipalities in South Africa with a population of about 177,577 people (Statistics South Africa, 2011). The majority of this predominantly rural local municipality is under traditional authority on Ingonyama Trust land, and the remaining land is commercial farmland (Msinga Municipality, 2018). The urban areas of Msinga are extremely small, with limited employment and growth opportunities. In this area, smallholder agricultural activities and other informal activities are the primary professions for the local residents, in an area that has medium to low potential for agricultural activities due to the local terrain, which is usually too rocky or too steep for annual cultivation (Mthembu, 2013; Msinga Municipality, 2018). Only 4.4% of the terrain in Msinga is regarded as arable land suitable for primary production (Mthembu, 2013). The main sources of livelihood strategies are widely off-farm and on-farm wages, government social grants, remittances, and cash income derived from land-based livelihoods. Msinga is classified, under the Köppen-Geiger climatic zones, as a “Cwa” climatic zone (Warm temperate climates with dry winters and hot summers) and the area receives an average annual rainfall of about 697 mm (Climate-Data.Org, 2020). In terms of disaster risk management – according to the Msinga Local Municipality: Disaster Management Plan 2015 –the ability of the municipality to implement comprehensive, integrated disaster management is low. Msinga is continuously affected by extreme weather events, mainly drought, which intensifies runaway fires (Msinga Local Municipality, 2015).

Umbumbulu is located approximately 45 km south-west of the centre of Durban, 19 km from the Indian Ocean. It forms part of the eThekwini Metropolitan Municipality. The area is mostly rural with small pockets of peri-urban settlements. Most of Umbumbulu is under traditional authority that is headed by an *iNkosi* (Chief) who influences local institutions and affairs such as tribal courts, land tenure and allocation of land rights, and a local government representative council (Ortmand and Machete, 2003). The majority of smallholder households in Umbumbulu are female-headed. The Umbumbulu area is classified under the Köppen-Geiger climatic zones, as a “Cfa” climatic zone (Water temperate climates with nu dry season and hot summers) (Climate-Data.Org, 2020). Its terrain is undulating with a mean annual rainfall of about 1 020 mm occurring mainly during the summer (Buthelezi, 2010). It is located in a moist coastal hinterland region with the climate favourable for growing a wide range of crops (Garikai, 2014). Crops grown include *amadumbe (taro)*, potatoes, beans, maize, sugarcane, bananas, chillies and peanuts. Maize, legumes and potatoes are the dominant crops grown (Buthelezi, 2010; Garikai, 2014).

Richmond local municipality is located within the uMgungundlovu District Municipality, approximately 40 km from Pietermaritzburg (the capital of KZN). This local municipality has a population of 65,793 people (Statistics South Africa, 2011). The majority of the population resides in rural areas, further away from the capital city, making it difficult to access. This is especially apparent during rainy seasons due to the muddy gravel roads that constrain market access and may also result in the area being neglected in terms of social services provision. Similar to Msinga and Umbumbulu, rural landholding is predominantly governed through the traditional authority. The area is classified under the Köppen-Geiger climatic zones, as a “Cfb” climatic zone (Warm temperate climates with no dry season and warm summers) and receives about 852 mm of rain per year, with most rainfall occurring during mid-summer (Climate-Data.Org, 2020; Weatherbase.com, 2020). The unemployment rate is very high in Richmond; approximately 77% of households earn less than R1500 a month (Pillay, 2016). In comparison to Msinga and Umbumbulu, Richmond has relatively better service provision, with 89.7% of households having access to electricity (South African Government, 2018). The area produces timber, sugarcane, poultry and dairy goods (Richmond Municipality, 2015). The rural Richmond population have limited and uneven access to resources, resulting in smallholder farmers farming in degraded conditions with minimal resources and knowledge (Pillay, 2016).

### 2.2 Research design

A qualitative research approach (Flick, 2004) was applied for this study. In this research, we aimed to understand different adaptation pathways adopted by the selected rural communities to cope with drought. Focus group discussions and individual interviews were conducted on a purposively selected study population.

### 2.3 Data and methods

Smallholder farmers were the targeted population for this study. These were defined as farmers who farm for subsistence and basic income on agricultural lands less than 1 ha within marginal settings. The study included crop-farming smallholders, livestock-farming smallholders, and smallholders who were involved in mixed farming activities (participating both in crop production and livestock farming). Following the rationale as described by Tongco (2007), we adopted a purposive sampling technique to target diverse representation of smallholder farming communities within the selected study areas.

#### 2.3.1 Data collection processes and tools

Focus group discussions and individual interviews were used as data collection tools to gather primary data from the three research sites. This allowed the researchers to get “first-hand” perspectives and attitudes related to the subjects studied (Kumar, 2010). Ethical clearance for this research was obtained from the London School of Hygiene and Tropical Medicine's research ethics committee, and the Humanities & Social Sciences Research Ethics committee of the University of KwaZulu-Natal. The Consolidated Criteria for Reporting Qualitative research (COREQ) were followed for reporting the results of this study. Written informed consent was obtained from all participants before data collection.

Focus group discussions (FGD) were conducted using pre-tested topic guides. Also, semi-structured individual interviews (IDIs) were conducted to explore personal experiences. They were later combined with the FGD data to produce a more comprehensive understanding of the mechanisms and to increase the depth of the study findings (Tierney and Clemens, 2011). Participant selection was conducted using a purposive sampling method: a community worker contacted village elders and community leaders, who approached people from local communities. Study volunteers met two inclusion criteria: 1) being a local smallholder farmer resident in the study area, and 2) being over the age of 18.

Focus group participants were separated into small groups (between 5 and 9 participants) according to gender and age to ensure that everyone was comfortable sharing their experiences. IDIs were carried out with participants who had not been part of the FGDs to understand personal views of the issues being studied. Two facilitators conducted each FGD and IDI at a suitable and quiet location. Both FGDs and IDIs were conducted in isiZulu, the local language, to allow the discussion to flow naturally. The FGDs lasted approximately an hour and the IDIs 40 min.

The FGD facilitators and interviewers were fluent in both English and isiZulu. They received one-week of training to familiarise them with the objectives, topic guides and questioning techniques. A pilot study was conducted before data collection, the results of which are not included in this study. Feedback on the pilot study was used to improve and finalise the topic guides. All FGDs and IDIs were recorded on password-protected Olympus VN-541PC recorders and stored securely. The recordings were subsequently transcribed into English ad verbatim.

Seven FGDs were conducted: six FGDs were held among female participants and one among male participants. All FGD's were conducted in Msinga, given the good relationships that were established with the community, which enabled this form of data collection. The FGD's therefore represent the adaptation strategies of Msinga. Nine IDIs were conducted: seven with female participants and two with male participants. The IDIs were conducted in Richmond and Umbumbulu, with only one IDI taking place in Msinga. Community leadership in Richmond and Umbumulu stated that IDI's were more appropriate than FDG's, as it was challenging to set up group discussions. Male and female participants were separated to reduce bias and allow participants to participate in the discussions freely.

#### 2.3.2 Data processing

Thematic analysis was conducted on the translated scripts utilising NVivo 12 for Mac, based on grounded theory (Strauss and Corbin, 1998). Themes were identified as they emerged from the participant FGD and IDIs. Firstly, all transcripts were read in full to gain an overall understanding of the data. Subsequently, each transcript was re-read to identify general categories, which were then systematically coded. The codes were combined or contrasted to develop and interpret overarching themes, generating a network of associations. At the end of this process, theme categories were reviewed to ensure they represented the data well. Where necessary themes were merged or split into sub-categories. Key themes that emerged from the FGD and IDIs were discussed between two researchers (PH and PS), and any disagreements were resolved in discussion with a third researcher (TM).

## 3 Results

A total of 57 people voluntarily participated in the study (F = 50 and M = 7), in July 2018. The participants were over 18 and were classified as smallholder farmers (Table 1, Table 2). The median age was 50 years old.

**Table 1:** Location and characteristics of focus group discussion participants.

Code Name	Date Conducted	District of Study Site	Age Range (years)	Total Number in FGD	Sex	Av. No in household	Occupation of the group
*FGD 1*	05/07/2018	Msinga	32–65	7	All Female	7	Smallholder farmer
*FGD 2*	05/07/2018	Msinga	32–76	9	All Female	8	Smallholder farmer
*FGD 3*	July 05, 2018	Msinga	30–80	8	All Female	6	Smallholder farmer
*FGD 4*	12/07/2018	Msinga	30–62	6	All Female	8	Smallholder farmer
*FGD 5*	12/07/2018	Msinga	25–52	7	All Female	5	Smallholder farmer
*FGD 6*	12/07/2018	Msinga	30–67	6	All Female	6	Smallholder farmer
*FGD 7*	12/07/2018	Msinga	46–63	5	All Male	5	Smallholder farmer

**Table 2:** Location and characteristics of individual interview participants.

Code Name	Date Conducted	District of Study Site	Age (years)	Sex	No. in household	Occupation
*IDI1*	12/07/2018	Msinga	47	Male	7	Smallholder farmer
*IDI2*	19/07/2018	Richmond	53	Female	8	Smallholder farmer
*IDI3*	19/07/2018	Richmond	60	Female	6	Supervisor at a shop (smallholder household)
*IDI4*	19/07/2018	Richmond	48	Female	5	Smallholder farmer
*IDI5*	20/07/2018	Umbumbulu	56	Female	11	House wife (smallholder household)
*IDI6*	20/07/2018	Umbumbulu	63	Female	6	Smallholder farmer
*IDI7*	20/07/2018	Umbumbulu	62	Male	4	Smallholder farmer
*IDI8*	20/07/2018	Umbumbulu	45	Female	5	Smallholder farmer
*IDI9*	20/07/2018	Umbumbulu	40	Female	5	Smallholder farmer

Three principal themes emerged during the thematic analysis of the data:

### 3.1 Theme 1 – perceived effects of droughts on the local food system and diets

This theme illustrates the main pathways through which drought was perceived to affect food security in the three rural areas. An altered food environment to access ingredients for daily meals – for example, by visiting a supermarket – was frequently mentioned as a preventative action to avoid becoming food insecure after drought-related harvest failures. A recurrent theme was the vulnerability of poor rural smallholder communities to climate variability. The severe impact droughts were reported to have affected food availability, food prices, diets and food security. The respondents' main perceived change in diets was a shift from vegetable-based diets to starch-based diets because of their inability to produce any fresh vegetables during drought periods.

### 3.2 Theme 2 – current coping strategies [short-term emergency responses] implemented

This theme summarises the strategies currently practised by rural communities and the perceived effects on the health, social and economic systems of the communities. All strategies implemented by the respondents were coping strategies [i.e. short-term or emergency responses]. Within this theme strategies were categorised into two sub-themes: direct (food-related) and indirect (economic) coping strategies.

### 3.3 Theme 3- enablers for successful [mid-to long-term] adaptation

This theme explored the adaptive [i.e. mid-to long-term] strategies that would allow effective adaption to climatic events. Furthermore, it synthesises the respondents’ views on effective measures or interventions that would increase their resilience and enable them to adapt successfully to increased drought frequency and duration and ensure food security year-round.

#### 3.3.1 Theme 1 –perceived effects of droughts on the local food system and diets

Drought was perceived to reduce smallholder farmers' ability to produce enough and diverse food for their families or additional crops to sell. A reduction in the production – and hence consumption – of vegetables and animal-sourced food (ASF) was frequently mentioned as one of the negative consequences of drought periods. Respondents reported that droughts reduced the quantity and deteriorated the quality of several commonly grown vegetables. Also, it affected their ability to generate additional income from selling surplus produce. Furthermore, respondents mentioned that droughts made their land unsuitable for some types of vegetables and increased risk of premature livestock death. Together, this was reported to have resulted in a higher dependency on shops to access foods, which – in many cases – was said to substantially alter the diets of people living in rural smallholder communities. The main dietary change reported was a shift from predominantly vegetable-based to predominantly starch-based diets and reduced ASF consumption. People expressed concern that diets, in times of prolonged droughts, were less nutritious than years of good yield and were perceived to affect their overall health negatively.

There were also some concerns about their daily food basket costs when respondents had to access a shop for their daily meals. Government grants enabled people to buy some additional supplies in retail supermarkets, but these were rarely enough in drought times.

*“Normally we do not buy them* [tomatoes and onions]*, or any other vegetable we cannot grow anymore, as it is pointless for us to spend the little we have on small quantities that are overpriced.”* FGD 3(5) - female

*“The people who kept livestock here, cattle, chickens, goats, have lost their animals as there was not enough water and food for them, so they died. So, we do not eat as much meat and animal products as we used to, because now we have to buy it from the shops and it is very expensive.“-* FGD 3(3) - female

The shift away from vegetable consumption was hypothesised to be accelerated by preferential choices of the younger generation, who were reported to prefer more processed and “western” foods above a predominantly vegetable-based diet.

The reduced capacity to keep livestock also affected the food system by reducing manure availability, resulting in an increased need for fertilisers to grow crops, which was often unaffordable.

*“Because we have no livestock, we are forced to be more reliant on fertilisers instead of organic manure. We cannot do anything much about this since almost all the cattle died during the drought.”* - FGD 3 (4) - female

Alongside this, respondents mentioned an increase in crop disease and pests due to climate variability, which destroyed crops and resulted in a reliance on pesticides. Pesticides were often inaccessible due to price and knowledge, so were unable to be used.

*“ …. now we have noticed new diseases and pests that we are not used to, and we're not sure where they come from. For example, now that its winter, these pests are still in our fields.” –* FGD 1(2) - female

#### 3.3.2 Theme 2 – current short-term coping strategies

**Direct (food-related) coping strategies;** in the most acute stages of a food-insecure period, respondents reported purchasing cheaper, less nutritious and more starch-based foods. They frequently mentioned only buying the *“basics”* in these acute periods. Starchy foods were perceived as more filling for relatively lower costs and had a longer shelf-life than fresh products, so they could be bought in bulk - to reduce price - and kept for longer. As a result of these coping strategies, people expressed concern that diets during drought periods were unhealthy.

*“We are reliant on the shops even though these are expensive so usually we buy less of a variety and just stick to the basics – maize meal, we buy more of this to keep us full instead of spending money we can't afford to spend on vegetables.”* FGD 3(4) –female

*“What we used to eat in the past is more nutritious compared to what we eat now.” FGD 2(1) - female*

Reports of respondents being more tired and becoming dizzy whilst working in the field were prominent – especially in Msinga. Few mentioned diseases in the community, but they did not know whether these were linked to the coping strategies implemented and subsequent dietary shifts. Reducing portion sizes – but not meal frequency – was also a commonly mentioned coping strategy - particularly in Msinga - where people mentioned hunger and tiredness a lot more. The last meal of the day was most likely to be skipped or reduced, as in the respondents’ opinion *“hunger never bothers a sleeping person". FGD 4(2).* Skipping a meal was reported to be rare; however, it occurred sporadically among women who prepared the meals: they explained that it is common practice for female family cooks to make sure everyone else is served first before they serve themselves.

[in response to the effects of reduced portion sizes on their family] *…” It makes us more tired because we don't get full and we still need to work in the fields.”* - FGD 7(4) - male

[In response to how reducing meal potions affects their family] …. . *“My family, it leads them to feel more tired, and they become hungry quicker, and they feel dizzy more often in the day …”* – FGD 3(3) - female

Some respondents reported holding on to small-stock to build in insurance in case of a further prolonged drought, referred to as the *“dark days”.* The respondents explained that most people would not keep livestock for consumption, but “reserve” them to sell in periods of acute food insecurity. In the meantime, they would benefit from the manure produced. Due to their relatively low cost and relatively high resistance to droughts, respondents kept goats and chickens in preference to other animals.

“I have my own chickens and goats, but we can't just kill and eat them all the time, because then we would have none left. I need to keep my animals in case there is an emergency. Maybe one day I need money for food or to help my family, and I need to sell a goat or a chicken, they are my security.” – IDI 1 (Male).

A strong sense of community was reported across all sites, including sharing of food among neighbours when needed. However, many respondents agreed that - due to extreme and acute food insecurity at times - there was not always an opportunity to share food.

**Indirect (economic) coping strategies;** In times of extreme droughts and decreased harvests, participants reported a strong focus on finding alternative ways to increase income and ensure their families could afford to purchase food. Activities to generate income varied across the study sites and included taking children out of school to work, begging, and borrowing money from loan sharks (local informal moneylenders), family or friends. Respondents in Msinga were more likely to borrow from loan sharks, whereas in Richmond and Umbumbulu residents were more likely to borrow money from neighbours or family members.

Due to low adaptive capacity, the prolonged use of coping strategies seemed to apply additional stress to the residents – especially in areas where drought was more severe; it reduced their resilience and ability to respond adequately. The continued stress and worry of not being able to feed your family, were distracting, had detrimental effects on the community's mood and mental health and affected productivity and motivation in the communities.

#### 3.3.3 Theme 3 - Enablers to successful (mid-to long-term) adaptation

**Irrigation and water supply –** Respondents reported mostly on the immediate and short-term emergency responses that they could carry out independently within their families and communities. They also had strong views on factors that would enable longer-term adaptation with external help from local governments and other non-governmental organisations. In Richmond and Umbumbulu, some adaptation measures were already in place – though not always fully functioning – whereas in Msinga respondents expressed the desire for a broader and more comprehensive rollout of adaptation measures to all villages.

Well-functioning irrigation systems - or emergency water services - were reported to play a pivotal role in continuing food production and, hence, preventing food insecurity in poor rural areas. A government system to assist drought-affected people was mentioned as the primary alleviation mechanism.

*“We are also fortunate in this area, because we have irrigation water. We source the water from the nearby Tugela River, using irrigation pipes …. Because of this, we are able to plant few crops in winter.”* – FDG 2(5) - female

*“Our lives depend on the irrigation system, without it we would not survive!”* –IDI1 - male

However, not all sites had fully functioning irrigation infrastructure. Systems were unable to provide sufficient water year-round due to dropping water tables. Furthermore, maintenance issues-such as frequent blockage – resulted in an interrupted water supply to farmers.

*“The water levels have started to decrease, and the irrigation pipes and nearby stream are now full of mud. Sometimes we need to drain the mud to get irrigation water.”* –FDG 7(3) – male

Interrupted water supply was reported to be most prominent in the planting season, potentially damaging the growth of crops from the start. Respondents suggested several adaptations to improve their irrigation systems, but feel they lack the capacity to act.

*“It would help if we could* have *a machine to drain the mud from the stream. A lot of women have been injured trying to drain mud from the stream and irrigation pipes that supply water to the fields.”* – FDG 7(4) - male

In Richmond and Umbumbulu the government was reported to routinely provide municipal water trucks in low rainfall times. This water was used for drinking, washing and but also irrigating smallholder and homestead crops.

*“Water becomes scarce during drought, and we are unable to do anything. We rely mainly on the municipal water trucks that deliver water to the community in times of drought”* – IDI9 - female

**Increased use of agricultural inputs** and agricultural advancement – Respondents frequently mentioned the potential longer-term benefit of pesticides and drought tolerant cultivars (DTCV) that could improve harvests in times of drought stress and reduce the vulnerability of crops to pests. The participants stated that pests were more frequent nowadays due to warmer weather and the lack of frost that would normally kill them off; crops grown in drought are under higher strain – pests increase this strain and further reduce yield and quality. Limited knowledge of effective use of pesticides and DTCV was mentioned as a barrier to using them in their fields. The awareness of the need to use them was explicitly stated across all sites. Still, without training on practical use, the respondents perceived pesticides to be of high expense but little benefit. Agricultural inputs were frequently mentioned as long-term adaptation measures and were perceived as highly suitable for the local context.

*“We also need drought-resistant crop cultivars and help knowing which ones to plant that can grow in the drought.”* – FGD 4(7) - female

*“Our biggest challenge is money for buying and remembering the names of the chemicals that would help us. We're not sure whether these chemicals are supposed to be applied before planting, during planting or when the maize has started growing.“*- FGD 7(1) - female

**Improved storage capacity for crops and water -** In Masinga, the respondents reported opportunities to strengthen resilience and adaption through storage technologies. The respondents mentioned that surplus crops are produced during harvest season, but they are unable to store them. As a result, the excess yield has to be used - and in times of low yield - they have no backup and have to rely on shops and other coping strategies, such as changing their diet. Rainwater storage facilities were also mentioned as an alternative source of water that would increase resilience - respondents would be able to collect water during rainy seasons to use in times of drought.

Finally, comparing the three study sites revealed some apparent differences in perspectives between people residing in areas more severely affected by droughts (Masinga & Umbumbulu) and people living in the less affected Richmond site. The latter seemed to receive more government assistance and aid during the more acute stages of the drought, such as regular water supply by trucks - compared to Umbumbulu and Masinga. This resulted in some slightly more positive comments and views from respondents in Richmond.

*“There aren't any barriers [to improved resilience and adaptation]. It's a matter of being creative. For example, rearing chickens is good because they do not get affected by drought and during drought conditions, one can sell chickens and use the extra income.”* – IDI 2 - female

## 4 Discussion

Using rural KwaZulu-Natal as a case study, this study identified pathways through which periods of drought affect food systems and diets among smallholder communities in South Africa. The study explored the perceived dietary impacts of drought in smallholder communities and smallholder farmer views on various short- and long-term strategies to respond to drought. Short-term coping (or emergency) strategies were widely practised, whilst several longer-term adaptation strategies were reported not to be accessible even though widely perceived to be appropriate and potentially useful in the smallholder context. The two settlements closer to urban nodes - Richmond and Umbumbulu – described lower reduction in meal frequency and ASF consumption than Msinga - the deep rural area – however, adaptive capacity was still low, and short-term coping strategies were the only options. These findings highlight the need for assistance in developing and implementing long-term adaptation strategies to drought in smallholder communities. Further research needs to be conducted on the potential health impacts that could arise from long-term dietary change.

Agricultural production is sensitive to climate variability and change that triggers water and food-related crises especially among marginal smallholder farming communities who depend on climate-sensitive agriculture for household and community-based food security (Pelser, 2001; Twongyirwe et al., 2019; Kom et al., 2020; Payus et al.*, 2020**)*. The main reported consequence of drought-related food insecurity in this study was reduced consumption of non-starchy vegetables and ASF, which was substituted by higher consumption of starch-based foods. This mirrors findings of previous research on drought-affected areas, reporting a decrease in dietary diversity whilst starch intake increased during times of drought (Ravindranath et al., 2005; Pandey et al., 2007; Chauvin et al., 2012; McGee et al., 2014) and could be explained by the relatively high vulnerability of vegetables to water deficits compared to starchy crops (Chauvin et al., 2012; Ochieng et al., 2016, 2017).

Reduced access to diverse diets is a significant contributor to undernutrition in sub-Saharan Africa (Luchuo et al., 2013; Myers et al., 2017). Vegetable and ASFs are vital sources of essential vitamins and minerals (Murphy and Allen, 2003; Randolph et al., 2003; Miller et al., 2013). A predominately starch-based diet may be adequate in energy, but is unlikely to be nutritionally adequate (Brinkman et al. 2009). The dietary shifts reported in this study could, therefore have the potential to increase the risk of micronutrient deficiencies in rural smallholder communities if continued long-term. This highlights how local coping strategies could lead to mal-adaption if not well-informed from different perspectives looking at the same problem. Local adaptation strategies tend to be always informed by the availability of resources and capacity at a local level, access to climate information, level of education and gender roles (Kom et al., 2020) and given the composition of most rural communities in South Africa, these are always likely to fail. Therefore, climate-change adaptation strategies must be designed with local communities following multiple perspectives approaches, incorporating local perspectives and scientific perspectives to avoid maladaptation, enhancing community resilience (Kom et al., 2020). Micronutrient deficiencies are associated with low adult work productivity, impaired physical and mental development, disease and poor pregnancy outcomes (Popkin, 2006; Brinkman et al., 2009; Arimond et al., 2010; Miller et al., 2013; Wang et al., 2014; World Health Organisation WHO, 2016; Alissa and Ferns, 2017; Ochieng et al., 2017).

Plant physiological processes could partly explain the relatively higher vulnerability of vegetables to drought. Vegetables are succulent and commonly consist of more than 90% of water. Therefore, water stress, particularly at critical growth stages, may drastically reduce yield and vegetable quality (Chatterjee and Solankey, 2014; Tuomisto et al., 2018). However, historically, plant breeding for food security has mainly focussed on calorie provision, rather than dietary diversity. Hence, this has contributed to the limited advancements in the development of drought resistant-vegetable varieties. Research on the development of drought-tolerant varieties has mainly focused on producing enough food from a caloric intake perspective and has concentrated on starch-based crops and staple foods (Daryanto et al., 2017; Tuomisto et al., 2018).

The evidence base and development of drought-tolerant fruit and vegetable varieties are much scarcer. However, these crop groups could play a vital role in improving public health nutrition in a sustainable way if incorporated, by policymakers and local municipalities, to local adaptation strategies (Mabhaudhi et al., 2019; Kom et al., 2020). An increase in research on the potential of drought-tolerant and nutrient-dense traditional leafy vegetables such as amaranth, moringa and wild mustard (Abukutsa-Onyango, 2010; Chivenge et al., 2017; Mabhaudhi et al., 2019) that are suitable for production under low input agricultural systems, which typify South Africa's rural landscape (Tesfay et al., 2016; Maseko et al., 2017), especially during harsh environmental conditions, has been noted and should inform local policy recommendations and adaptation strategies. Crops, such as amaranth, moringa and wild mustard could become an important resource for CC adaptation in rural South Africa – specifically for smallholder farmers - who rely on subsistence produce to supplement diets (Statistics South Africa, 2017a; Mabhaudhi et al., 2019). However, a paucity of information describing their agronomy and water use, as well as a lack of production guidelines have previously been cited as bottlenecks to their promotion (Chivenge et al., 2017; Mabhaudhi et al., 2015).

Lower ASF consumption has often been shown to benefit health and the environment (Westhoek et al., 2014; Springmann et al., 2016; Silva and Fischer, 2017). However, in the case of rural smallholder farmers, ASF's play a vital role in providing essential macro and micronutrients and increased consumption can improve nutrient adequacy of diets and reduce the risk of nutritional deficiencies (Murphy and Allen, 2003; Zhang et al., 2016; Grace et al., 2018; Headey et al., 2018). In a rural smallholder setting, owning livestock goes beyond providing food security; it acts as a financial buffer in times of hardship and plays a significant social and cultural role (Upton, 2004; Mahlobo, 2016). During drought, heat-stress alongside reduced fodder and water availability can lead to increased off-take rates (Udmale et al., 2014; McKune et al., 2015), reduced productivity, and increased vulnerability to disease and in severe cases mass livestock deaths (IPCC, 2012). Research into drought-resistant breeds of livestock shows that indigenous breeds and goats are more drought-resistant than exotic breeds (Darcan and Silanikove, 2018).

Keeping drought-tolerant species such as goats and more drought-resistant indigenous breeds could reduce the loss of livestock in times of drought and has been highlighted in the literature as an effective adaptive measure to mitigate the implications of drought (Nyamushamba et al., 2016; Scholtz et al., 2016; Darcan and Silanikove, 2018). Selling livestock and reducing ASF and vegetable consumption are still short-term strategies and the absence of well-planned, feasible, and contextualised long-term adaption techniques across all rural areas is evident. Droughts form a substantial challenge for ensuring adequate micro and macronutrient intake in rural South African smallholder communities. It is crucial for the diets and health of millions of rural inhabitants – predominantly smallholder farmers – that adequate adaptation strategies are developed and implemented by appropriate governmental and community-based actors to address this problem and ensure community resilience to different climate-related shocks (Kom et al., 2020).

**Fig. 2:**
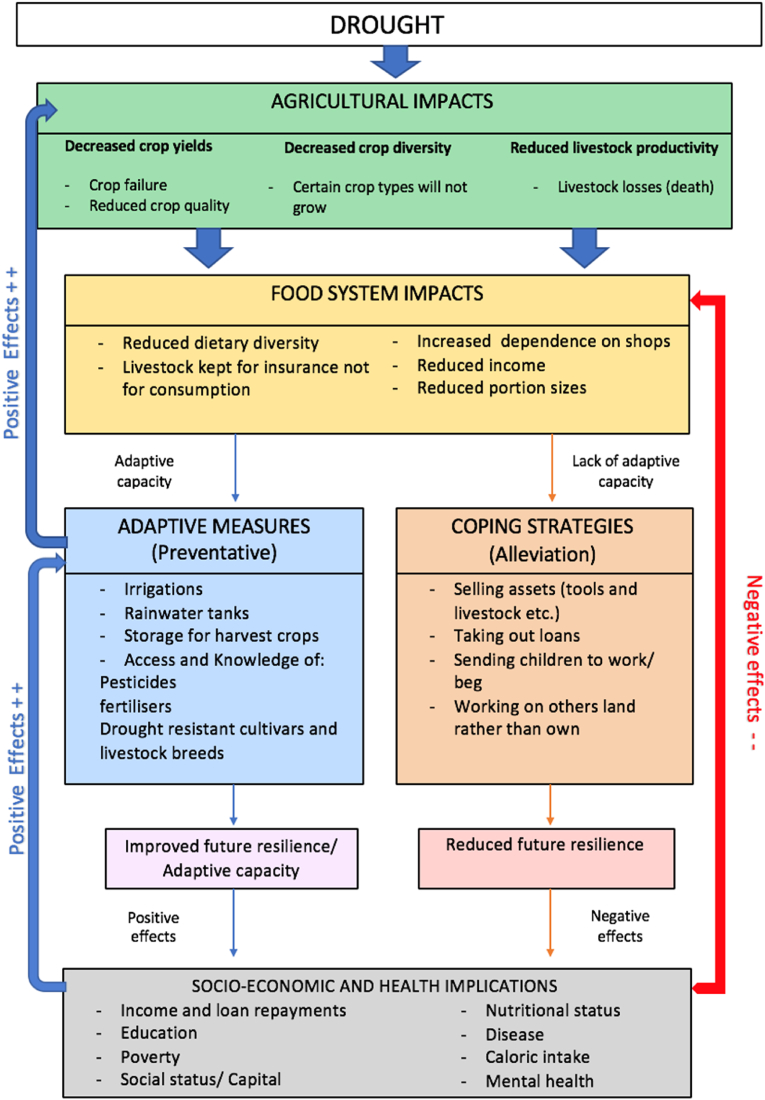
Framework summarising the concepts, variables and relationships involved in the potential effects of drought on smallholder food systems and the impacts of short-term coping strategies and long-term adaptive measures. This figure is formed using the themes and relationships between concepts identified in this study and previous literature and empirical data on this subject-discussed in this paper.

The respondents all reported engaging in short-term coping strategies; they frequently felt they did not have the capacity or resources to implement long-term adaption measures, even though they were aware of them. This highlights the low adaptive capacity of the smallholder communities in marginal and vulnerable settings. Misinformed coping strategies enable communities to survive short-term, but lead to a chain of events from mal-adaption to increased risk of food insecurity, negative health impacts, poor socio-economic outcomes and reducedadaptive capacity in the long-term – creating a cycle that is hard to break (IPCC, 2012; Ashraf and Kumar, 2013; Warner and Geest, 2013). A single coping or adaption strategy may not be effective on its own, but can play a vital role in complementing other strategies (Vermeulen et al., 2013; Wamsler and Brink, 2014). Coping and adaption can be closely connected – there are synergies and trade-offs, all of which are dependent on the context (Wamsler and Brink, 2014). The participants suggested several context-specific adaptive measures that would allow smallholder populations to adapt and recover from climate variability and improve long-term adaption to climate extremes and food security (Fig. 2).

The longer-term adaptation measures comprise stable smallholder production in times of drought and climate variability. This involves addressing both the direct (lack of water) and indirect (e.g. higher vulnerability to pests and reduced yield) impacts of droughts. Inputs should be useable and accessible to all drought-affected communities to have the most effective impact on adaption capacity (Basdew and Mafongoya, 2017). Supporting smallholder farmers by subsidising fertilisers, pesticides and DTCV and different livestock breeds, alongside providing education on practical use, has been shown to reduce the impact of drought, by increasing smallholder crop yield (Browne and Hendriks, 2007; Shiferaw et al., 2014). Rainwater harvesting and storage for surplus crops effectively aid farmers' survival and agricultural productivity in other drought-prone regions (Udmale, Ichikawa and Manandhar, 2014; Mdungela et al., 2015; Lindoso et al., 2018), as well as irrigation technology (Solh and Ginkel, 2014). Irrigation, however, has significant trade-offs with water availability for other uses when used as an adaption strategy (Rey et al., 2017) and when used alone can still leave communities in vulnerable positions during times of drought (Pandey et al., 2007; Vermeulen et al., 2013; Department of environmental affairs, 2018).

Another adaptation strategy could evolve around the diversification of crops and livestock, which has been shown to contribute to diversified diets through both subsistence and income-generating pathways – and may be an essential strategy for improving diets and nutrition outcomes in low- and middle-income countries (Jones, 2017). Agricultural diversification also acts as a risk management tool, protecting against climate extremes and price inflations and contributing to biodiversity and sustainable land management in smallholder farming (Food and Agriculture Organization of the United Nations, 2012; Jones, 2017; Rajendran et al., 2017). Many other adaptation strategies could be formulated. However, it is vital to always study and understand determinant drivers that affect local smallholder farmer’ adaptation pathways for well-designed and robust planning and successful implementation of adaptation methods at a local level (Kom et al., 2020).

In many cases, coping strategies achieve short-term survival because they are often not well-designed for long term, sustainable outcomes. They can come with trade-offs and can lead to maladaptation, causing increasingly adverse socioeconomic and health outcomes - making communities more, rather than less vulnerable to drought (Fig. 2). This framework was produced to summarise the concepts, variables and relationships involved in the effects of drought on smallholder food systems; produced using previous literature, the perceived consequences of drought prevalent in this study and relationships between these concepts. This included the role of short-term coping and longer-term adaptation strategies and the subsequent socioeconomic and health outcomes. Highlighting local knowledge and the opportunities and leverage points that could be created by engaging the local community in the co-development of adaptation strategies and incorporating indigenous knowledge to improve adaptive capacity and response to drought in smallholder communities in policy making processes is essential.

Studies that focused on a combination of adaptation strategies concluded that adaptive capacity could be further improved when several leverage points are addressed simultaneously (Ravindranath et al., 2005; Boko et al., 2008; Vermeulen et al., 2013). This study highlighted several leverage points where interventions could be employed to increase adaptive capacity long-term in rural smallholder farmers, and are illustrated in the framework (Fig. 2). Timely and adequate drought adaptation measures would also address other public health issues beyond nutrition such as the effect of drought on mental health, whichwas a sub-theme that emerged in this study and has been frequently reported elsewhere (Coêlho et al., 2015).

## 5 Strengths and limitations

The qualitative methodology of this study allowed perceptions, experiences and decision-making processes of smallholder farmers to be explored in-depth. This approach is critical for developing practical and contextualised future policies (Tierney and Clemens, 2011). The interviewers all spoke the native language fluently and were from Zulu backgrounds. This enabled rapport to be more easily formed, reducing response bias by increasing engagement, resulting in more in-depth, thoughtful, and honest answers (Holbrook et al., 2003).

However, the results of this study should be interpreted in the context of certain limitations. Qualitative research can be very subjective and dependent on the fieldworkers' ability facilitating the interviews, and respondent validation was not conducted. Extensive training and de-briefs after IDIs and FGDs were put in place to minimise this limitation. The recruitment of participants via purposive sampling and the contextual nature of adaption strategies means that this study's results cannot be extended to broader populations.

## 6 Conclusion

This study was conducted among a small group of rural smallholder farmers in KwaZulu-Natal, South Africa. The coping strategies and adaptive measures recorded in this study may be specific to these study sites, but increasing climate variability is a global threat, particularity to rural smallholders. With drought episodes predicted to become increasingly severe and frequent, understanding the strategies implemented by rural smallholder communities in the face of drought is crucial in developing future effective and context-specific policies.

The smallholder communities in this study respond to drought by changing their dietary habits in ways that may compromise health outcomes in the long-term. However, no consumption data or nutritional status assessment was recorded in this study. Therefore, further information on consumption and nutritional status related to the perceived changes in crop production and consumption, due to drought, is needed to fully assess environmental change's potential impact on public health nutrition (Belesova et al., 2020).

The respondents’ knowledge of long-term adaption measures was clear, however, they were not able to implement these long-term strategies without the support of government or other actors, and without further knowledge about the best strategies to adopt in each particular context. Further research into the most practical combination of adaption measures and ways of implementing them in rural smallholder communities is vital in improving adaptive capacity, and mitigating maladaptation and trade-offs created by short-term coping strategies. Also, considering the importance of vegetables to public health alongside their susceptibility to drought, research into drought-tolerant vegetables is an important research area alongside the promotion of production and consumption of traditional leafy vegetables which are mostly drought and heat stress tolerant, as well as nutrient-dense.

Although smallholder farmers produce a small percentage of food at a national level, they play a vital role in achieving household and local rural food security and combined, contribute substantially to global food security and should not be forgotten. Co-produced knowledge, which integrates scientific knowledge with local knowledge and practice, will lead to more sustainable and context relevant outcomes in this important sector, leading to long-term adaptation in the face of climate variability and climate change, and improved health and wellbeing.

## Research data related to this submission

There are no linked research data sets for this submission. The following reason is given: Data will be made available on request.

## Author contributions

PH: Conceptualization; Data curation; Formal analysis; Investigation; Methodology; Resources; Software; Validation; Visualization; Roles/Writing - original draft.

MN: Project administration; Visualization; Roles/Writing - original draft.

WG: Investigation; Writing - review & editing.

CS: Methodology; Writing - review & editing.

KQ: Methodology; Writing - review & editing.

AD: Roles/Writing - original draft; Funding acquisition; Supervision.

TM: Conceptualization; Data curation; Resources; Roles/Writing - original draft; Supervision;: Project administration;

PS: Conceptualization; Data curation; Methodology; Resources; Software; Validation; Visualization; Roles/Writing - original draft; Funding acquisition; Supervision; Project administration;

## Declaration of competing interest

The authors declare that they have no known competing financial interests or personal relationships that could have appeared to influence the work reported in this paper.
